# Improved exercise performance and increased aerobic capacity after endurance training of patients with stable polymyositis and dermatomyositis

**DOI:** 10.1186/ar4263

**Published:** 2013-08-13

**Authors:** Li Alemo Munters, Maryam Dastmalchi, Abram Katz, Mona Esbjörnsson, Ingela Loell, Balsam Hanna, Maria Lidén, Håkan Westerblad, Ingrid E Lundberg, Helene Alexanderson

**Affiliations:** 1 Rheumatology Unit, Department of Medicine, D2:01, Karolinska Institutet, Karolinska University Hospital, SE-171 76, Stockholm, Sweden; 2 Rheumatology Unit, Department of Physiotherapy, D2:07, Karolinska University Hospital, SE-171 76, Stockholm, Sweden; 3 Muscle Physiology, Department of Physiology and Pharmacology, Karolinska Institutet, SE-171 77 Stockholm, Sweden; 4 Department of Health Management, School of Health Sciences, Ariel University, Ariel 40700, Israel; 5 Department of Laboratory Medicine (LABMED), Division of Clinical Physiology, C188, Karolinska Institutet, SE-141 86, Stockholm, Sweden; 6 Rheumatology Unit, Department of Medicine, Område 4, Sahlgrenska University Hospital, SE- 413 45, Gothenburg, Sweden; 7 Rheumatology Unit, Department of Medicine, 30B, Uppsala University Hospital, SE-751 85, Uppsala, Sweden; 8 Department of NVS, Division of Physiotherapy, D3 Fack 23400, Karolinska Institutet, SE-141 83 Huddinge, Sweden

## Abstract

**Introduction:**

This randomized, controlled study on patients with polymyositis or dermatomyositis was based on three hypotheses: patients display impaired endurance due to reduced aerobic capacity and muscle weakness, endurance training improves their exercise performance by increasing the aerobic capacity, and endurance training has general beneficial effects on their health status.

**Methods:**

In the first part of this study, we compared 23 patients with polymyositis or dermatomyositis with 12 age- and gender-matched healthy controls. A subgroup of patients were randomized to perform a 12-week endurance training program (exercise group, *n *= 9) or to a non-exercising control group (*n *= 6). We measured maximal oxygen uptake (VO_2 _max) and the associated power output during a progressive cycling test. Endurance was assessed as the cycling time to exhaustion at 65% of VO_2 _max. Lactate levels in the *vastus lateralis *muscle were measured with microdialysis. Mitochondrial function was assessed by measuring citrate synthase (CS) and β-hydroxyacyl-CoA dehydrogenase (β-HAD) activities in muscle biopsies. Clinical improvement was assessed according to the International Myositis Assessment and Clinical Studies Group (IMACS) improvement criteria. All assessors were blinded to the type of intervention (that is, training or control).

**Results:**

Exercise performance and aerobic capacity were lower in patients than in healthy controls, whereas lactate levels at exhaustion were similar. Patients in the exercise group increased their cycling time, aerobic capacity and CS and β-HAD activities, whereas lactate levels at exhaustion decreased. Six of nine patients in the exercise group met the IMACS improvement criteria. Patients in the control group did not show any consistent changes during the 12-week study.

**Conclusions:**

Polymyositis and dermatomyositis patients have impaired endurance, which could be improved by 12 weeks of endurance training. The clinical improvement corresponds to increases in aerobic capacity and muscle mitochondrial enzyme activities. The results emphasize the importance of endurance exercise in addition to immunosuppressive treatment of patients with polymyositis or dermatomyositis.

**Trial registration:**

ClinicalTrials.gov: NCT01184625

## Introduction

The idiopathic inflammatory myopathies, including polymyositis (PM) and dermatomyositis (DM), are characterized by muscle weakness and poor muscle endurance, especially in proximal muscles [1,2]. The mechanisms that lead to muscle weakness are not fully clarified but both immune-mediated and non-immune-mediated mechanisms are thought to be involved in the disease process and the latter mechanisms include endoplasmic reticulum (ER) stress, hypoxia, autophagy and mitochondrial pathology [3-5]. Despite recommended treatment with high doses of glucocorticoids and immunosuppressive agents, few patients can regain their former muscle performance and the reason for this is not fully understood [6,7].

Abnormalities in energy metabolism has been observed in inflammatory myopathies, including PM and DM [8]. Patients with established PM/DM display fewer oxidative slow-twitch type I muscle fibers and lower aerobic capacity compared to healthy controls [9-11]. Thickened endothelial cells, reduced capillary blood supply and low total number of capillaries may impair oxygen transport to the muscle fibers in inflammatory myopathies [12,13]. Accordingly, up-regulation of hypoxia-related proteins, such as vascular endothelial growth factor (VEGF) and hypoxia-inducible factor-1 (HIF-1), has been observed in muscle of patients with inflammatory myopathies [13,14].

Traditionally, physical exercise has not been recommended to patients with PM/DM [6]. However, recent exercise studies in patients with established PM/DM revealed benefits and safety [6,15], with a potential to prevent muscle loss due to muscle inflammation, physical inactivity and systemic glucocorticoid treatment [16,17]. For instance, a resistance exercise program resulted in modulations of gene expression transcripts associated with inflammation (down-regulation), fibrosis (down-regulation) and aerobic metabolism (up-regulation) [18]. However, available studies have limitations and it is not possible to recommend one exercise program over another, indicating a need for randomized controlled trials [6,15].

The major objective of this randomized, controlled, multicenter study was to determine whether endurance exercise can improve muscle function in patients with PM/DM in a chronically stable disease phase. We tested the following hypotheses:

1. Compared to age- and gender-matched healthy controls, PM/DM patients display impaired performance during endurance exercise due to reduced aerobic capacity and muscle weakness (that is, an inability to fully activate the contractile machinery). To test this hypothesis, patients and healthy controls performed endurance exercise and we assessed performance, aerobic capacity, heart rate, muscular lactate concentration and self-reported exertion level.

2. Endurance training improves exercise performance in PM/DM patients by increasing muscle aerobic capacity. To test this hypothesis, patients were randomized into an exercise group (EG), performing a 12-week endurance training program, and a non-exercising control group (CG). Endurance exercise performance, muscular lactate concentration, aerobic capacity and mitochondrial capacity in muscle biopsies were assessed before and after the intervention period.

3. Endurance training has a general beneficial effect on the health status of PM/DM patients. The disease activity was assessed before and after the training period according to the International Myositis Assessment and Clinical Studies Group (IMACS) improvement criteria.

## Methods

### Study design

In the first part of the study we compared patients with PM/DM with age- and gender-matched healthy controls. The controlled part of the study was a hypothesis-driven randomized controlled trial evaluating effects of a supervised 12-week endurance exercise program compared to a non-interventional control group.

### Patients and healthy controls

Patients (*n *= 23) were recruited from 2007 to 2011 from three rheumatology units in Sweden: Karolinska University Hospital, Stockholm; Sahlgrenska University Hospital, Gothenburg; and Uppsala University Hospital, Uppsala, Sweden. Inclusion criteria: a) diagnosis of definite or probable PM or DM [19,20]; b) age ≥18 years; c) duration since diagnosis more than six months; d) exercising one time or less a week; e) stable medication for at least one month. Exclusion criteria: a) severe heart or lung conditions; b) severe osteoporosis; c) not being able to exercise.

Twelve healthy controls (HC) matched for age, gender and physical activity level were identified among each patient's non-genetic, related family members and friends and health care professionals. Inclusion criteria: a) age ≥18 years; b) exercising one time or less a week. Exclusion criteria: a) chronic musculoskeletal disorder; b) severe osteoarthritis or osteoporosis; c) severe heart or lung conditions. All participants gave informed consent to participate in the study, which was approved by the local ethics committees at Karolinska University Hospital, Sahlgrenska University Hospital and Uppsala University Hospital. The study followed the World Medical Association's 2008 Declaration of Helsinki.

### Aerobic capacity

Ergometer cycle maximal oxygen uptake (VO_2 _max) tests were performed by patients and HC at baseline and by patients after the 12-week intervention (training or control) period. Different cycling protocols were used for patients and HC because a previous study showed approximately 50% lower VO_2 _max in patients with PM/DM compared to HC [11]. Thus, the patients started at 30 to 40 W and the required power output was increased with 10 W every minute, while the HC started at 50 W and the increase was 15 W every minute. The VO_2 _max was determined by incremental cycling to exhaustion. Pulmonary minute ventilation and gas exchange (O_2 _uptake and CO_2 _output) were measured breath by breath (Sensor Medics, V max 229™, Yorba Linda, CA, USA). The system was calibrated for airflow and gas exchange before each test. VO_2 _max was defined as the highest O_2 _uptake rate measured during the test and is expressed as l min^-1 ^and as ml min^-1 ^(kg body weight)^-1^; the power performed (in W) at the time of VO_2 _max was recorded. At VO_2 _max, we also measured the respiratory exchange ratio (RQ) defined as the ratio between the exhaled CO_2 _and the inhaled O_2_. Electrocardiography was used to continuously follow the heart rate and the maximum heart rate was recorded. At the end of the test we recorded the self-reported ratings of peripheral exertion (lower extremity) and central exertion on the Borg RPE scale (6 to 20) [21].

### Cycling endurance test

Cycling endurance tests were performed by patients and HC at baseline and by patients after the 12-week intervention (training or control) period. Cycling was performed at a power requiring 65% of the VO_2 _max obtained in the baseline aerobic capacity test (see above) and was continued until exhaustion. The tests were supervised by the same physical therapist throughout the study. At exhaustion, self-reported peripheral and central exertions were recorded and the heart rate was measured.

### Lactate measurements

The extracellular concentration of lactate was measured *in vivo *with microdialysis. Two thin catheters with a 3 cm long membrane with a diameter of 0.5 mm (CMA 63) were inserted in the *vastus lateralis*, 2 cm apart, under local anesthesia [22]. A reference catheter was inserted in subcutaneous abdominal fat tissue. The microdialysis experiments were performed in patients and HC at baseline and in patients also after 12 weeks intervention (training or control) by the same two physicians, nurse and physical therapist throughout the study. The microdialysis probes were perfused with a physiological solution at a flow rate of 1 μl min^-1^. Water soluble substances in the interstitial fluid diffuse across the semipermeable dialysis membrane and enter the perfusate, which was collected in small vials. The cycling endurance test (see above) started after an 80-minute equilibration at rest, during which one vial of perfusate per catheter was collected every 20 minutes. After the endurance test, subjects rested for 60 minutes while vials with perfusate were collected every 20 minutes. Analysis of lactate concentration was made directly in a Clinical Microdialysis Analyzer (ICSUS, CMA, Solna, Sweden). Vials collected from the *vastus lateralis *muscle during the 20 minutes directly after cycling to exhaustion showed the highest lactate concentrations and the mean lactate concentration in vials obtained from the two catheters at this time interval was used in subsequent assessments.

### Muscle biopsies and measurements of mitochondrial enzyme activities

Muscle biopsies were taken from the *vastus lateralis *muscle under local anesthesia using a semi-open technique [23]. Biopsies were taken from the patients before and after the 12-week +/- endurance training. Due to technical reasons, biopsies were only taken from patients who were recruited at the Karolinska University Hospital and at the Uppsala University Hospital. We measured the activities of two mitochondrial enzymes, citrate synthase (CS) and β-hydroxyacyl-CoA dehydrogenase (β-HAD), which reflect the mitochondrial volume to cell volume ratio and the capacity for fatty acid β-oxidation, respectively [24,25]. Muscle biopsies were freeze dried, dissected free of non-muscle constituents and weighed. Tissue was then homogenized with ground glass homogenizers in ice-cold buffer (HxB) consisting of 50 mM KH2PO4; 1 mM EDTA, 0.05% (vol/vol) Triton X-100, pH 7.5. The homogenate was centrifuged at 1,400 × g for one minute (4°C). Aliquots of the supernatant were analyzed for CS and β-HAD with standard spectrophotometric techniques [26,27]. Enzyme activities were assayed at room temperature (approximately 22°C) under conditions that yielded linearity with respect to extract volume and time (data not shown). Protein content was measured in the supernatant with the Bradford assay (Bio-Rad Laboratories AB, Sundbyberg, Sweden and activities were adjusted for protein content.

### Clinical outcome measures

Assessment of disease activity was performed using the proposed preliminary consensus core measures developed for PM/DM patients by IMACS [7,28]. The core measures include: patients and physicians global disease activity assessments recorded on a Visual Analogue Scale (VAS, 0 to 100); Manual Muscle Testing-8 (MMT (muscle strength), 0 to 10 in eight muscle groups); Health Assessment Questionnaire (HAQ (limitation in daily activities), 0 to 3); measurement of the blood serum activity of the muscle enzyme creatine phosphokinase (s-CPK, μcat l^-1^); and assessment of the degree of disease activity in six organ systems other than skeletal muscle using the myositis intention to treat activity index (MITAX, 0 to 1). We used the proposed core measures and the assessment of disease activity in the skeletal muscle system rated on VAS (0 to 100). [28-30]

Damage was measured by Myositis disease Damage Index (MDI) proposed by IMACS, which involves clinical assessment of the degree of disease damage in 11 organ systems and the physician global damage tool on a VAS (0 to 100) [28,29,31].

The clinical assessment of each patient was performed by the same experienced physician before and after the 12-week intervention period and the assessor was blinded to the type of intervention, (that is, training or control).

### Endurance exercise training

**Figure 1:**
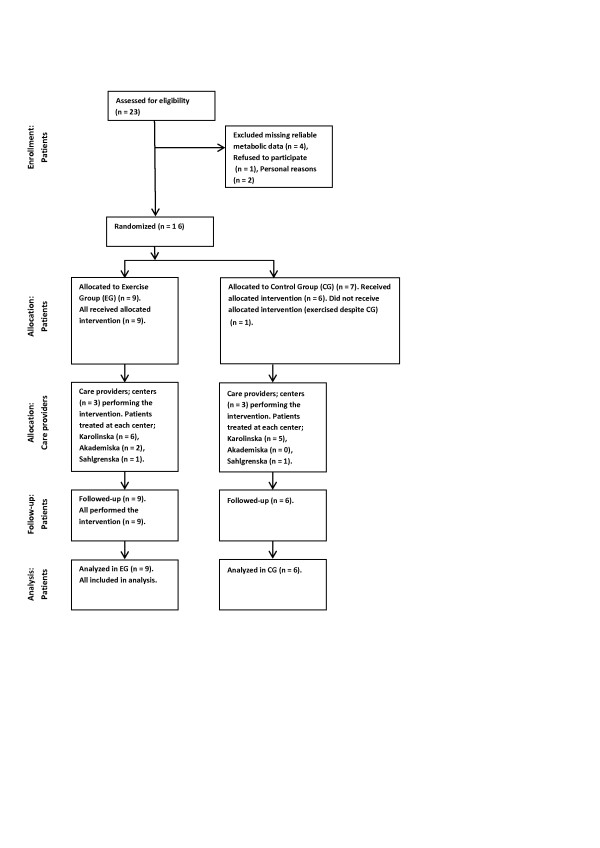
**Flow diagram for patients according to the CONSORT randomized, controlled trials of non-pharmacologic treatment**.

A subgroup of the PM/DM patients was randomized, using a randomization list, either to an exercise group (EG) or a control group (CG) (Figure 1). An independent nurse who was responsible for the randomization then contacted the exercise coach at each center to inform about group allocation. The EG performed an approximately one-hour exercise program three times a week for 12 weeks. The exercise program consisted of five-minute warm-up cycling at 50% of VO_2 _max, which was followed by up to 30 minutes of more intense cycling where the intensity was gradually increased during the training period aiming at 70% of VO_2 _max. This was followed by 20 minutes of muscular endurance exercise engaging both the upper and lower limbs at 30 to 40% of one voluntary repetition maximum. The program ended with a cool-down and stretch. At each participating center, the EG was supervised by the same physical therapist. The patients in the CG were instructed not to change their physical activity level during the 12-week intervention period, but they were invited to perform the same endurance exercise program after the intervention period.

All patients used exercise diaries to register their physical activities during the 12-week intervention period. All nine patients randomized into the EG participated in the training program as scheduled according to their exercise diary. Seven patients were randomized to the CG; however, one started on her own initiative another endurance training program; thus six patients were analyzed in the CG.

### Statistical analyses

Data are presented individually, as mean (± SD) or as median (range). Student's unpaired *t*-test was used to detect differences between patients and HC. For evaluation of the effect of endurance training, measurements performed before and after the intervention period were compared using Student's paired sample *t*-test. The significance level was set to *P *<0.05. A power test was performed assuming changes in physiological measurements after endurance training being 30 ± 20% of the control value. With a power of 0.80 and an alpha of 0.05, this gives a sample size of six. Based on this, we aimed for nine patients in the EG, but some analyses were performed with *n *= 7 (VO_2 _max measurements) or *n *= 3 (mitochondrial enzyme activities).

We used the proposed definition by IMACS to define improvement in disease activity during the intervention period [32]. To be a responder, a patient should improve ≥20% in ≥50% of IMACS core measures with no more than two worse by ≥25%, which could not include measures of muscle function (MMT). Improvement in muscle function and exercise performance to the 12-week endurance exercise was evaluated and presented for the individual patients. A responder was defined as improvement by ≥20% in MMT and/or cycling time at 12-week follow-up compared to baseline [33].

Statistical analyses were made using Statistica 10.0 (StatSoft, Inc., Tulsa, Ok, USA) or SigmaStat 3.1 (Systat Software, Inc., Point Richmond, CA, USA).

## Results

### Patients versus healthy controls

Twenty-three PM or DM patients and 12 HC participated in the initial assessments and their respective demographics are presented in Table 1. The patients were in a stable disease phase with unchanged medication for at least one month before inclusion in the study and had a low disease activity.

**Table 1:** Baseline data for patients with polymyositis and dermatomyositis and healthy controls.

	Patients* n = 23 Median (range)	Healthy controls* n = 12 Median (range)	Exercise group*¤ *n *= 9 Median (range)	Control group*¤ *n *= 7 Median (range)
**Sex** **(female/male), n**	17/6	9/3	8/1	6/1
**Diagnosis** **(PM/DM), n**	11/12	NA	4/5	3/4
**Age, years**	58 (37 to 77)	51 (31 to 71)	60 (48 to 72)	58 (42 to 77)
**Duration since** **diagnosis, years**	7 (1 to 33)	NA	8 (1 to 13)	7 (2 to 33)
**DMARD treatment** **(GC/MTX/AZA/RITUX/MMF/CsA), n**	13/9/5/2/1/1	NA	5/3/3/0/1/0	5/2/2/1/0/1
**Daily GC dose, mg**	5 (1 to 10)	NA	5 (1 to 8)	2 (2 to 5)
**MMT-8, 0 to 80**	75 (42 to 80)	NA	76 (42 to 79)	73 (57 to 77)
**HAQ, 0.0 to 3.0**	0.5 (0.0 to 1.8)^1^	NA	0.5 (0.0 to 1.4)^1^	0.5 (0.4 to 1.8)
**CPK, μcat/l**	2 (1 to 62)^3^	NA	2 (1 to 62)^1^	2 (1 to 4)^1^
**Physician global** **disease activity** **VAS, 0 to 100**	4 (8 to 16)^2^	NA	4 (0 to 14)^1^	2 (0 to 10)^1^
**Patient global** **disease activity** **VAS, 0 to 100**	39 (0 to 88)^1^	NA	24 (0 to 72)^1^	42 (3 to 77)
**MITAX, 0 to 1.0**	0.1 (0.0 to 0.2)^2^	NA	0.1 (0.0 to 0.2)	0.1 (0.1 to 0.2)^1^
**Muscle disease activity, VAS, 0 to 100**	2 (0 to 8)^2^	NA	2 (0 to 8)^1^	2 (0 to 7)^1^
**Physician global damage VAS, 0 to 100**	18 (0 to 62)^2^	NA	22 (7 to 62)^1^	22 (10 to 49)^1^
**Muscle damage** **VAS, 0 to 100**	11 (0 to 39)^2^	NA	10 (0 to 39)^1^	14 (9 to 31)^1^

*Data in median (range) if not stated otherwise. ^¤^, Patients with reliable lactate levels at baseline were randomly assigned to an exercise group or a control group. AZA, Azathioprine; CPK, creatine phosphokinase (normal values <2.5 μcat/liter in women, <3.0 μcat/liter in men); CsA, Cyclosporine A; DMARD, disease modifying anti-rheumatic drugs; GC, Glucocorticoids; HAQ, Health Assessment Questionnaire; MITAX, myositis intention to treat activity index; MMF, Mycophenolate mofetil; MMT, manual muscle test; MTX, Methotrexate; NA, not assessed; PM/DM, polymyositis/dermatomyositis; RITUX, Rituximab; VAS, visual analogue scale. ^1^, One missing datum; ^2^, Two missing data; ^3^, Three missing data. *, No statistical difference in any of the variables between groups; patients vs HC and EG vs CG assessed by Mann-Whitney U-test (*P *<0.05).

**Figure 2:**
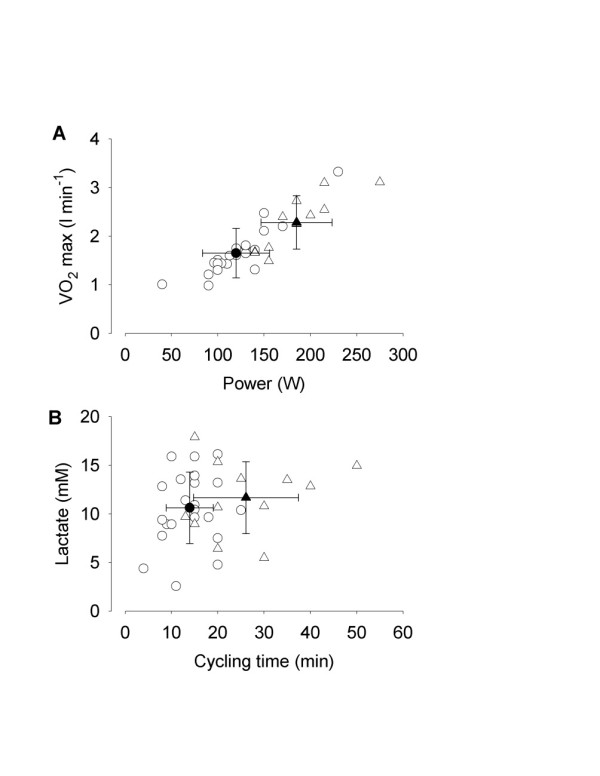
**Polymyositis and dermatomyositis patients have lower aerobic capacity and endurance exercise performance than healthy controls**. **A**. VO_2 _max *vs*. the corresponding power output obtained in the progressive bicycle test. **B**.Concentration of lactate in *vastus lateralis *muscle dialysate after cycling vs. cycling time in the endurance test performed at the same relative work load in all individuals. Individual data from 23 patients (open circles) and 12 healthy controls (open triangles). Mean data (± SD) from patients (filled circle) and healthy controls (filled triangle) are also shown.

We used a progressive ergometer cycle test to measure VO_2 _max and the corresponding power output. PM/DM patients had an approximately 25% lower VO_2 _max (*P *<0.01) and an approximately 35% lower power output (*P *<0.001) than HC (Figure 2A and Table 2). On the other hand, the RQ at VO_2 _max, the maximum heart rate, and Borg ratings of peripheral and central exertion were similar in patients and HC (Table 2).

**Table 2:** Lower maximal oxygen uptake and endurance performance in myositis patients compared to healthy controls.

	Patients *n *= 23 Mean ± SD	Healthy Controls *n *= 12 Mean ± SD	Unpaired t-test
**VO_2 _max test**			

VO_2 _max (l min^-1^)	1.65 ± 0.51	2.28 ± 0.55	*P *<0.01
VO_2_, max/body weight (ml min^-1 ^kg^-1^)	22 ± 5	32 ± 6	*P *<0.01
Power at VO_2 _max (W)	120 ± 36	185 ± 38	*P *<0.001
Respiratory exchange ratio(RQ = CO_2_/O_2_)	1.1 ± 0.1	1.2 ± 0.1	n.s.
Maximum heart rate (min^-1^)	160 ± 15	173 ± 16	n.s.
Borg rating (6 to 20):			
Peripheral exertion	19 ± 1	19 ± 1	n.s
Central exertion	17 ± 3	18 ± 2	n.s.

**Endurance cycling test**			

Time to exhaustion (minute)	14.0 ± 5.1	26.1 ± 11.3	*P *<0.001
Lactate (mM)	10.6 ± 3.6	11.7 ± 3.7	n.s.
Percentage maximum heart rate	91 ± 1	96 ± 1	n.s.
Borg rating (6 to 20):			
Peripheral exertion	19 ± 1	18 ± 2	n.s
Central exertion	17 ± 1	18 ± 2	n.s

n.s., Non-significant; VO_2 _max, maximal oxygen uptake; endurance cycling test, time cycling to exhaustion performed with a constant power, which in each individual was set to requiring 65% of baseline VO_2 _max; Borg rating, rating of exertion at end of VO_2 _max test and at the end of the endurance cycling test; Lactate, concentration of lactate measured in muscle *vastus lateralis *with microdialysis directly after the endurance cycling test.

An endurance cycling test was performed with a constant power, which in each individual was set to that requiring 65% of VO_2 _max; that is, the mean power during this test was markedly lower in patients than in HC. The lactate concentration in the *vastus lateralis *muscles was measured with microdialysis after this endurance test. The cycling time to exhaustion was approximately 45% shorter in the patients than in HC (*P *<0.001), despite the fact that patients cycled at a lower power (Figure 2 and Table 2). On the other hand, the lactate concentration, the percentage of max heart rate and Borg ratings of peripheral and central exertion were similar in patients and HC at the end of the endurance test (Table 2).

### Effects of a 12-week endurance exercise program

A subgroup of the patients (*n *= 15) with reliable metabolic marker data was included in the analysis of the effect of a 12-week intervention period. All patients tolerated the exercise program well and no adverse events were noted.

**Figure 3:**
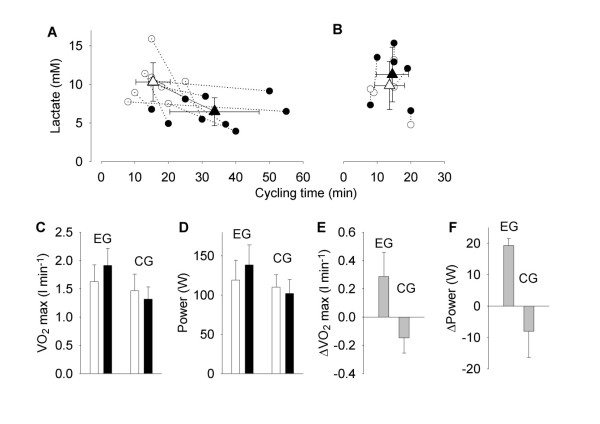
**Exercise and aerobic capacity are increased in myositis patients after 12 weeks endurance training**. **A**. Individual data of the concentration of lactate in muscle dialysate after cycling vs. cycling time (at the same absolute work load for each subject) obtained in nine patients before (open circles) and after (filled circles) endurance training. Mean data (± SD) before (open triangle) and after (filled triangle) training are also shown. **B**. Same as in A but for six patients in the control group (no training). Mean data (± SD) of VO_2 _max (**C**) and the corresponding power (**D**) before (white bars) and after (black bars) the 12-week intervention period in the exercise group (EG, *n *= 7) and the control group (CG, *n *= 5). **E **and **F **show mean data (± SD) of the change in VO_2 _max and power after *vs*. before the intervention period in individual subjects.

All patients in the EG increased their time to exhaustion in the cycling endurance test by ≥20% and mean data show an approximately doubled cycling time (*P *<0.01; Figure 3A and Table 3). Moreover, lactate measured after the endurance test was about 35% lower after than before the training period (*P *<0.01). Conversely, the heart rate at the end of the cycling test and Borg ratings of peripheral and central exertion were similar before and after the training period (Table 3). The patients in the CG, who kept a stable physical activity level, showed no consistent changes in either cycling performance or lactate concentration during the 12 weeks of the study (Figure 3Band Table 3). Their heart rate and Borg ratings at exhaustion were also similar before and after the 12 weeks.

**Table 3:** Improved aerobic capacity and endurance performance after 12 weeks of endurance exercise in myositis patients.

	Before Mean ± SD	After Mean ± SD	Paired t-test
**Exercise group (EG)**			

**Endurance cycling test (*n *= 9)**			
Time to exhaustion (minutes)	15.4 + 5.1	33.7 + 13.3	*P *<0.01
Lactate (mM)	10.3 ± 2.5	6.5 ± 1.8	*P *<0.01
Percentage maximum heart rate	88 ± 8	88 ± 5	n.s.
Borg rating (6 to 20):			
Peripheral exertion	19 ± 1	17 ± 2	n.s.
Central exertion	19 ± 1	17 ± 2	n.s.
**VO_2 _max test (*n *= 7)**			
VO_2 _max (l min^-1^)	1.63 ± 0.30	1.91 ± 0.30	*P *<0.01
Power at VO_2 _max (W)	119 ± 25	138 ± 26	*P *<0.001
**Mitochondrial enzyme activities (*n *= 3)**			
Citrate synthase (μmol (g dw^-1^) min^-1^)	42.9 ± 2.0	76.8 ± 1.4	*P *<0.001
β-hydroxyacyl-CoA dehydrogenase (β-HAD; μmol (g dw^-1^) min^-1^)	33.5 ± 6.5	50.1 ± 1.9	*P *<0.05

**Control group (CG)**			

**Endurance cycling test (*n *= 6)**			
Time to exhaustion (minutes)	13.7 ± 4.5	14.5 ± 4.8	n.s.
Lactate (mM)	9.9 ± 3.1	11.3 ± 3.5	n.s.
Percentage maximum heart rate	97 ± 2	95 ± 3	n.s.
Borg rating (6 to 20):			
Peripheral exertion	18 ± 2	18 ± 3	n.s.
Central exertion	17 ± 2	17 ± 2	n.s.
**VO_2 _max test (*n *= 5)**			
VO_2 _max (l min^-1^)	1.46 ± 0.30	1.32 ± 0.21	*P *<0.05
Power at VO_2 _max (W)	110 ± 16	102 ± 18	n.s.
**Mitochondrial enzyme activities (*n *= 5)**			
Citrate synthase (μmol (g dw^-1^) min^-1^)	52.8 ± 7.3	50.3 ± 13.0	n.s.
β-hydroxyacyl-CoA dehydrogenase (β-HAD; μmol (g dw^-1^) min^-1^)	43.8 ± 10.1	36.7 ± 10.5	n.s.

n.s., Non-significant; VO_2 _max, maximal oxygen uptake; endurance cycling test, time cycling to exhaustion performed with a constant power, which in each individual was set to requiring 65% of baseline VO_2 _max; Borg rating, rating of exertion at end of VO_2 _max test and at the end of the endurance cycling test; Lactate, concentration of lactate measured in muscle *vastus lateralis *with microdialysis directly after the endurance cycling test.

Reliable VO_2 _max tests were performed in seven patients in the EG and in five patients in the CG (Figure 3C-F). VO_2 _max and power output at VO_2 _max were higher in all patients in the EG after the endurance exercise period and mean values increased by approximately 20% (*P *<0.01; Table 3). Conversely, VO_2 _max was slightly decreased in the CG after the 12 weeks, whereas the power output did not change (Table 3).

**Figure 4:**
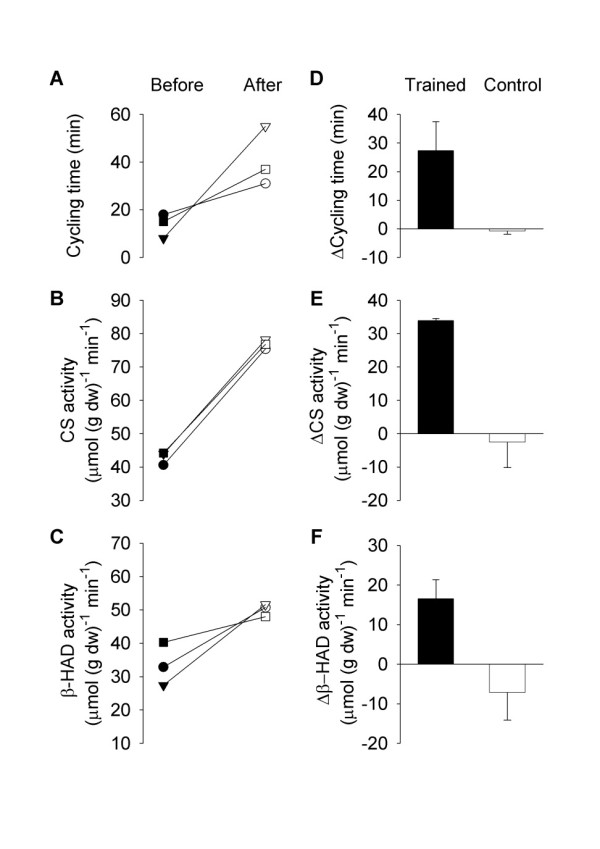
**Increased mitochondrial enzyme activities in muscles of myositis patients after 12 weeks of endurance training**. Individual data from three patients obtained before and after 12 weeks of endurance training for cycling time at the same absolute work load for each subject **(A) **and the activity of two mitochondrial enzymes, CS **(B) **and β-HAD **(C)**, in muscle biopsies from vastus lateralis muscle. **D-F**. Mean data of the difference after *vs*. before training (*n *= 3); data for five patients in the control group (no training) are also shown.

The improved cycling performance and VO_2 _max after training might be due to increased muscular mitochondrial respiration. To test this possibility, we measured the activities of two mitochondrial enzymes, CS and β-HAD, in muscle biopsies. All three EG patients tested had a major improvement in cycling performance (Figure 4A), CS activity (Figure 4B) and β-HAD activity (Figure 4C). Thus, mean data show significant increases in cycling performance (Figure 4D, P <0.05), CS activity (Figure 4E, P <0.001), and β-HAD activity (Figure 4F, P <0.05; Table 3). On the other hand, there were no significant changes in cycling time, CS activity or β-HAD activity in the CG (Figure 4D-F and Table 3).

Six out of nine patients in the EG were responders to the endurance exercise intervention according to the IMACS criteria (Table 4). Note that there was a slight discrepancy between IMACS responders (six out of nine) and responders in relation to muscle endurance (nine out of nine; see Figure 3A, Table 4). Conversely, only one out of six patients in the CG was improved according to the IMACS criteria (Table 4). This patient showed improvement in activity limitation (HAQ), physician's global disease activity and patient's global disease activity, whereas no improvement was observed in muscle endurance (cycling time) or muscle strength (MMT).

**Table 4:** Improved endurance performance and disease activity after 12 weeks of endurance exercise in myositis patients.

**Patient**	**Group**	**MMT** **0 to 80**		**HAQ** **0.00 to 3.00**		**s-CPK** **μcat/l**		**Physician's** **global disease activity** **VAS** **0 to 100**		**Patient's** **global disease activity** **VAS** **0 to 100**		**Mitax** **0.00 to 1.00**		**Cycling** **time minutes**	
	
	**EG/CG**	**0w**	**12w**	**0w**	**12w**	**0w**	**12w**	**0w**	**12w**	**0w**	**12w**	**0w**	**12w**	**0w**	**12w**
	
**A†**	EG	79	79	0.00	0.25	6.2	1.2	5	0	3	1	0.05	0.00	18	**31‡**
**B†**	EG	70	75	0.50	0.38	2.2	1.9	4	0	72	5	0.11	0.07	8	**55‡**
**C†**	EG	75	77	0.25	0.00	1.8	1.3	0	0	9	0	0.24	0.19	15	**37‡**
**D†**	EG	76	80	0.38	0.00	4.2	2.3	0	0	0	9	0.10	0.06	25	**30‡**
E	EG	42	**57‡**	NA	1.63	6.8	5.2	NA	3	NA	43	0.02	0.22	13	**20‡**
**F†**	EG	76	75	0.50	0.50	NA	NA	14	0	15	0	0.16	0.04	15	**50‡**
G	EG	71	76	0.38	0.38	1.5	2.0	3	0	32	11	0.00	0.00	10	**15‡**
H	EG	76	78	0.50	0.50	1.0	1.1	11	7	54	53	0.06	0.02	20	**40‡**
**I†**	EG	77	77	1.38	0.63	1.3	1.3	10	3	24	13	0.11	0.02	15	**25‡**
J	CG	77	77	0.38	0.63	1.6	3.1	0	0	44	26	0.19	0.11	9	10
**K†**	CG	72	74	0.38	0.00	1.8	1.8	1	0	13	5	0.06	0.10	15	15
L	CG	66	73	0.50	0.13	1.3	1.2	0	0	77	52	0.09	0.21	15	15
M	CG	75	70	0.50	0.38	3.7	1.9	3	5	20	44	0.14	0.13	20	20
N	CG	57	**75‡**	1.75	2.00	NA	NA	NA	5	59	69	NA	0.24	8	8
O	CG	75	72	0.63	0.88	1.2	1.6	10	6	39	59	0.13	NA	15	**19‡**

Individual results in myositis core set measures for disease activity and endurance performance (cycling time) before and after training (exercise group) or in the non-exercising control group. Data are analyzed by criteria for minimal clinical improvement set for patients with polymyositis and dermatomyositis [32]. 0 w, baseline assessment; 12 w, follow-up assessment; CG, control group (*n *= 6); EG, exercise group (*n *= 9); HAQ, health assessment questionnaire; MITAX, myositis intention to treat activity index; MMT, manual muscle test; NA, not assessed; s-CPK, creatine phosphokinase, (normal values <2.5 μcat/l in women, <3.0 μcat/l in men). Classification of improvement in disease activity measures: †, improved compared to baseline by ≥20% in ≥50% of measures with no more than two worsened by ≥25%, which cannot include MMT. ‡, improved muscle function (MMT/cycling time) compared to baseline by ≥ 20%.

## Discussion

In this study we report a substantially lower endurance exercise capacity in patients with PM/DM in a stable disease phase compared to healthy controls matched for age, gender and level of physical activity. We also show that 12 weeks of endurance training markedly improved endurance exercise performance in the patients and this improvement was accompanied by increased aerobic capacity. The training period also resulted in a general clinical improvement in the majority of patients.

In accordance with our first hypothesis, we show a decreased performance during endurance exercise in PM/DM patients compared to age- and gender-matched HC. The impaired performance in the patients was accompanied by a lower VO_2 _max and, hence, decreased aerobic capacity, which is also in agreement with our hypothesis. We also hypothesized that the patients' exercise performance would be limited by weakness and inability to adequately activate the exercising muscles. The results, however, show no difference between patients and HC in extracellular lactate levels, RQ values, experienced exertion level or percentage of maximum heart rate in the fatigued state; all these four measures would be lower if exercise ended prematurely due to improper muscle activation [34]. Nevertheless, the present cycling exercise requires relatively low muscle power output and exercises requiring a larger fraction of the available muscle force may well be limited by muscle weakness. For instance, such exercises like "walking up one flight of stairs" and "getting up from the ground/floor" have been reported as difficult to perform by patients with PM/DM [35,36]. Thus, in the present study patients with PM/DM show decreased performance during cycling endurance exercise and this impairment can be explained by reduced aerobic capacity, which is in agreement with a previous study [11]. Furthermore, we suggest that an important mechanism contributing to the reduced aerobic capacity and impaired endurance performance in the patients is a low mitochondrial capacity in muscle. Others have observed that a local inflammatory environment is associated with a mitochondrial dysfunction [37]. It is worth noting that the patients in the present study, as well as in previous studies, have been given conventional immunosuppressive treatment for at least six months, which consequently is not sufficient to regain full muscle endurance performance. In addition, long-term glucocorticoid treatment may cause mitochondrial dysfunction [38] and atrophy in skeletal muscle [39] and these deleterious effects may be aggravated when combined with low physical activity [40], which could be one explanation for persistent impaired performance in PM/DM patients after immunosuppressive treatment.

Our results show a marked improvement in endurance exercise performance in the PM/DM patients after 12 weeks endurance training, which is in agreement with our second hypothesis. This improvement could be explained by increased aerobic capacity after the training period as judged from a lower lactate level and an unchanged relative maximum heart rate in the fatigued state despite a longer cycling time; increased VO_2 _max and power output at VO_2 _max; and increased activity of the two mitochondrial enzymes CS and β-HAD. These findings of increased mitochondrial capacity by endurance training in patients with PM/DM are in accordance with previous studies showing improved aerobic capacity and up-regulated transcription of genes for proteins involved in aerobic metabolism in PM/DM patients exposed to a period of physical exercise [17,18,41]. It is worth noting that all patients exposed to the training program improved their exercise performance, that is, also patients with initially severely decreased muscle function and endurance capacity. Thus, endurance training is effective in PM/DM patients even when they are in a chronic stable disease phase with severe muscle dysfunction. It would be of great value to know if endurance training is beneficial also in an acute disease phase, but this is outside the scope of the present study.

In the present study, we measured extracellular lactate concentration in muscle after an individually determined cycling endurance test; that is, subjects cycled until exhaustion at a power requiring 65% of their VO_2 _max at baseline. The lactate concentration and, hence, the extent of anaerobic metabolism required, was similar in PM/DM patients and healthy controls at exhaustion, despite the fact that healthy controls cycled for longer times and at higher power outputs (see Figure 2). A markedly different picture would emerge if we instead would have used a fixed endurance test, that is, the same absolute power output and duration in both groups. The power output and duration tolerated by the PM/DM patients would then be rather mild for the healthy controls and markedly higher lactate levels would be expected in patients at the end of the test. In line with these assumptions, a recent case study showed markedly higher lactate levels after a fixed endurance test in a PM patient than in healthy controls [42]. Furthermore, after five weeks of endurance training, the PM patient had a lower lactate concentration at the end of the endurance test [42], which is in good agreement with our present results (see Figure 3).

In accordance with our third hypothesis, a majority (six out of nine) of the patients exposed to the training program improved concerning disease activity according to IMACS improvement criteria. In a previous seven-week resistance exercise study, two out of seven patients improved according to the same criteria [43]. These results indicate that endurance exercise, longer exercise duration, or a combination of both might be more beneficial than resistance exercise for PM/DM patients in a stable chronic disease phase.

One limitation in this study is that the median age of the HC group was seven years lower than the patients, which might exaggerate the differences between the two groups. However, it should be noted that there was no statistical difference in age between the two groups and they had a similar level of physical exercise. Another potential limitation is the relatively low number of patients in the EG and the CG. Since the patients included in this study were in a stable disease phase, no consistent changes during 12 weeks was expected in the CG and this is also what our results show. A power analysis showed that for physiological assessments we needed measurements from at least six subjects before and after the endurance exercise period. Nine patients were included in the EG, but all measurements were not successfully performed both before and after training in each subject. This limitation resulted in measurements of mitochondrial enzyme activities being clearly underpowered with only three patients in the EG. However, all three subjects showed clear increases in both CS and β-HAD activities after the training period (see Figure 4), which from a physiological perspective fits with the concomitant increases in aerobic capacity and cycling performance (see Figure 3). Finally, it would have been of interest to quantify a potential relationship between reduced disease activity and increased exercise performance in the EG after the training period. Unfortunately, the IMACS six-item core set does not consist of one total score and is, therefore, not suitable for quantifying relationships. However, we could demonstrate that all patients in the EG with reduced disease activity also improved in exercise performance (≥20 % increased cycling time).

## Conclusions

In conclusion, PM/DM patients show impaired endurance performance during cycling exercise due to reduced aerobic capacity. After 12 weeks of endurance training, PM/DM patients increased their mitochondrial enzyme activities in muscle, increased aerobic capacity, decreased disease activity and markedly improved exercise performance. These results emphasize that in order to improve muscle performance, PM/DM patients should be prescribed physical endurance exercise in combination with the recommended immunosuppressive treatment.

## Abbreviations

β-HAD: β-hydroxyacyl-CoA dehydrogenase; CG: control group; CS: citrate synthase; DM: dermatomyositis; EG: exercise group; ER: endoplasmic reticulum; HAQ: Health Assessment Questionnaire; HC: healthy controls; HIF-1: hypoxia-inducible factor-1; IMACS: International Myositis Assessment and Clinical Studies Group; MDI: myositis disease damage index; MITAX: myositis intention to treat activity index; MMT: Manual Muscle Testing; PM: Polymyositis; s-CPK: the blood serum activity of the muscle enzyme creatine phosphokinase; VAS: Visual Analogue Scale; VEGF: vascular endothelial growth factor; VO_2 _max: maximal oxygen uptake

## Competing interests

The authors declare that they have no competing interests.

## Authors' contributions

LAM coordinated the study, participated in the study design, performed intervention and microdialysis experiments, analyzed clinical and experimental data, and drafted the manuscript. MD participated in the study design and recruitment, and collected experimental and clinical data. AB performed and analyzed the mitochondrial enzyme activity experiments and assisted in the analyses of physiological results. ME and IL participated in the study design. BH and ML participated in recruitment and collected clinical data. HW analyzed experimental data and assisted in drafting the manuscript. IEL developed and planned the study design, collected experimental and clinical data, and participated in recruitment. HA developed and planned the study design and participated in analysis of clinical data. All authors participated in discussions and the finalizing of the manuscript and approved the final manuscript.
